# 589. Clinical Utility and Patient Characteristics Associated with Positive Whole Blood PCR for *Borrelia spp*. in an Endemic Area Using Novel High-Definition PCR^TM^ assay: A Case Series

**DOI:** 10.1093/ofid/ofad500.658

**Published:** 2023-11-27

**Authors:** Jasmine Mutti, Franklin Moore, Armando Paez

**Affiliations:** Baystate medical center, Ludlow, Massachusetts; Baystate Health, Springfield, Massachusetts; UMass Chan Medical School - Baystate, Springfield, Massachusetts

## Abstract

**Background:**

Current guidelines recommend antibody testing to detect acute borreliosis. Detectable antibody response takes about 2-3 weeks limiting its usefulness in early infection. The ChromaCode High-Definition PCR (HDPCR)™ Tick-Borne Pathogen Panel (TBP) is a research use only qualitative, real-time PCR assay for detection and identification of tick-borne pathogens including *Borrelia spp.* from whole blood samples.

**Methods:**

HDPCR™ TBP was put in clinical use after this was validated in the laboratory to identify *Anaplasma phagocytophilum, Babesia microti, Ehrlichia spp, Borrelia spp. (B. burgdoferi B. mayonii*, and *B. miyamotoi)*. Chart review was performed on all (+)*Borrelia spp.* from 8/1/22 to 12/31/22. Descriptive analysis was performed on each case. All data was captured and stored using RedCap.

**Results:**

Over a period of 5 months, 9 (0.5%) out of 1667 tested were (+) for *B. burgdorferi*; one (0.06%) was (+) for *B. miyamotoi*; out of these 6 cases had clinical information for review as described in Table 1. Mean age was 64 years; 4 were white and 2 were Hispanic. All 6 lived in western Massachusetts, and only 1 had a known history of tick bite. All had reported systemic symptoms at the time of testing. 3 had (+) *B. burgdorferi* IgM antibodies;1 case tested (+) PCR for *B. miyamotoi* but serology was not performed. 1 case was (+) for *B. burgdorferi* PCR 28 days after symptom onset and had babesiosis co-infection; 1 with *B. burgdorferi* mono-infection had a (+) PCR test on day 14 of symptoms. All 6 had symptom improvement after doxycycline treatment for 10-14 days.

Case description

**Figure:**
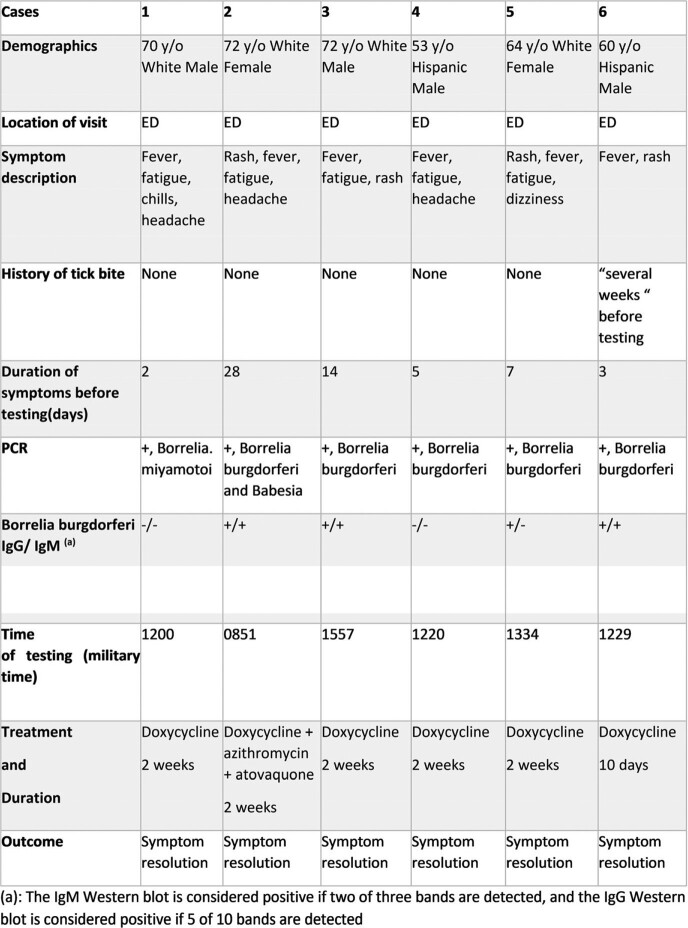

**Conclusion:**

HDPCR™ TBP for *Borrelia spp* is a low yield test, even in an endemic region. All (+) cases were symptomatic and clinically improved following doxycycline treatment supporting the test’s validity. Two cases had negative IgM antibody testing and diagnosis would be likely missed without PCR testing. None were immunocompromised including 2 with prolonged PCR positivity with 1 case having babesiosis co-infection. Study was limited only to 6 cases excluding analysis of other tick-borne infections as part of syndromic testing. Clinical usefulness and timing of HDPCR™ TBP to diagnose borreliosis and other tickborne illnesses need further study.

**Disclosures:**

**All Authors**: No reported disclosures

